# Evaluation of acute esophageal radiation-induced damage using magnetic resonance imaging: a feasibility study in mice

**DOI:** 10.1186/s13014-019-1396-8

**Published:** 2019-10-30

**Authors:** Pouya Jelvehgaran, Jeffrey D. Steinberg, Artem Khmelinskii, Gerben Borst, Ji-Ying Song, Niels de Wit, Daniel M. de Bruin, Marcel van Herk

**Affiliations:** 1 Department of Biomedical Engineering and Physics, Amsterdam UMC, University of Amsterdam, Meibergdreef 9, 1105 AZ Amsterdam, the Netherlands; 2 Department of Radiation Oncology, Amsterdam UMC, University of Amsterdam, Amsterdam, the Netherlands; 3 Department of Physics and Astronomy, Institute for Laser Life and Biophotonics Amsterdam, Amsterdam, the Netherlands; 4 Mouse Clinic for Cancer and Aging (MCCA) Imaging Unit, The Netherlands Cancer Institute (NKI), Amsterdam, the Netherlands; 5 Department of Radiation Oncology, The Netherlands Cancer Institute (NKI), Amsterdam, the Netherlands; 6 Department of Experimental Animal Pathology, The Netherlands Cancer Institute (NKI), Amsterdam, the Netherlands; 7 Department of Urology, Amsterdam UMC, University of Amsterdam, Amsterdam, the Netherlands; 8 Manchester Cancer Research Centre, Division of Cancer Sciences, Faculty of Biology, Medicine, and Health, University of Manchester, Manchester Academic Health Sciences Centre, Manchester, UK

**Keywords:** Esophagus, MRI, Radiation-induced damage, Radiation therapy

## Abstract

**Background:**

Thoracic and head and neck cancer radiation therapy (RT) can cause damage to nearby healthy organs such as the esophagus, causing acute radiation-induced esophageal damage (ARIED). A non-invasive method to detect and monitor ARIED can facilitate optimizing RT to avoid ARIED while improving local tumor control. Current clinical guidelines are limited to scoring the esophageal damage based on the symptoms of patients. Magnetic resonance imaging (MRI) is a non-invasive imaging modality that may potentially visualize radiation-induced organ damage. We investigated the feasibility of using T2-weighted MRI to detect and monitor ARIED using a two-phased study in mice.

**Methods:**

The first phase aimed to establish the optimal dose level at which ARIED is inducible and to determine the time points where ARIED is detectable. Twenty four mice received a single dose delivery of 20 and 40 Gy at proximal and distal spots of 10.0 mm (in diameter) on the esophagus. Mice underwent MRI and histopathology analysis with esophageal resection at two, three, and 4 weeks post-irradiation, or earlier in case mice had to be euthanized due to humane endpoints. In the second phase, 32 mice received a 40 Gy single dose and were studied at two, three, and 7 days post-irradiation. We detected ARIED as a change in signal intensity of the MRI images. We measured the width of the hyperintense area around the esophagus in all mice that underwent MRI prior to and after irradiation. We conducted a blind qualitative comparison between MRI findings and histopathology as the gold standard.

**Results/conclusions:**

A dose of 40 Gy was needed to induce substantial ARIED. MRI detected ARIED as high signal intensity, visible from 2 days post-irradiation. Quantitative MRI analysis showed that the hyperintense area around the esophagus with severe ARIED was 1.41 mm wider than with no damage and MRI-only mice. The overall sensitivity and specificity were 56 and 43% respectively to detect any form of ARIED. However, in this study MRI correctly detected 100% of severe ARIED cases. Our two-phased preclinical study showed that MRI has the potential to detect ARIED as a change in signal intensity and width of enhancement around the esophagus.

## Introduction

Advanced image-guided radiation therapy (IGRT) improves the clinical outcome of patients with thoracic and head and neck cancer. However, radiation therapy (RT) causes damage to healthy organs close to the tumor, such as radiation-induced damage to the lungs and esophagus when treating lung cancer [1, 2]. Esophageal toxicity can be classified as acute or late [3, 4]. Acute radiation-induced esophageal damage (ARIED) is often a dose limiting factor during lung cancer RT [2, 5–8]. Most patients undergoing lung RT are expected to develop ARIED because the esophagus is sensitive and often close to the tumor and/or involved lymph nodes [2, 7, 9, 10]. Concurrent chemo-RT with 60*–*66 Gy dose delivered in 6*–*7 weeks showed ARIED (grade > 3) in 21% of the patients [11]. Hyperfractionation in RT may increase the probability of ARIED (grade > = 3) to 45% of patients [12]. Moreover, patient who suffer from gastroesophageal reflux and other preexisting diseases on the esophagus may be more sensitive to develop ARIED [12]. In current clinical practice, ARIED is scored based solely on patient symptoms [13, 14]. ARIED can cause reduced food intake, nausea, dysplasia, odynophagia, anorexia, and other complications, which may interrupt treatment [3, 7, 13–17]. Treatment interruptions can affect tumor control [15, 17] and can likely be avoided if we can detect ARIED prior to patients developing symptoms and start countermeasures over time. In addition, knowing the exact location of ARIED is helpful for dose-response modelling. Hence, an in-depth understanding of ARIED can help to optimize IGRT planning, which may better control the tumor while reducing complications to nearby healthy regions. Various medical imaging modalities can visualize ARIED, such as white light endoscopy (WLE), positron emission tomography (PET) [18], and more recently, optical coherence tomography (OCT) [15, 17]. With the advent of magnetic resonance imaging (MRI) guided radiotherapy, we may be able to non-invasively detect and monitor ARIED.

MRI produces high-resolution images with good soft tissue contrast, which is ideal to distinguish organs from surrounding tissue. While MRI has been used to image the esophagus in a number of patient studies [19–22], there are no studies on imaging esophageal radiation-induced damage using MRI. ARIED manifests itself as morphological changes to the physical structure of the esophageal tissue [15]. Hence, we hypothesized that T2-weighted MRI could be used as a non-invasive imaging modality to visualize ARIED. Furthermore, our protocol was designed to evaluate whether there is a radiobiological sensitivity difference between the proximal and distal portions of the esophagus. In this two-phased study, we investigated the feasibility of MRI to visualize and monitor ARIED in vivo in mice. We performed qualitative and quantitative analysis of our MRI findings and compared our results with histopathology as the gold standard.

## Methods

### Experiments

We used specific-pathogen-free FVB female mice from a commercial vendor (Janvier, Le Genest-Saint-Isle, France). Our experimental protocols were approved by the local ethics committee and were performed in compliance with the guidelines of the European community (EUVD 86/609/ EEC) for the care and use of laboratory animals. Mice were housed in individually ventilated cages (IVCs) with filter tops, placing four to five mice per cage. Acidified drinking water and 4.0% fat mouse chow diet were available in all cages. According to the protocol, animals were euthanized at the end of the experiment using CO_2_ inhalation. The humane end-points to euthanize animals during the experiment were defined as a loss of 15% of the body weight and/or signs of major hair loss. During procedures mice were anesthetized using isoflurane (1.5% in O_2_ and air). Anesthetized mice were placed on a heating pad attached to the mouse bed to maintain their normal body temperature [15].

#### Pilot study (phase I)

**Fig. 1:**
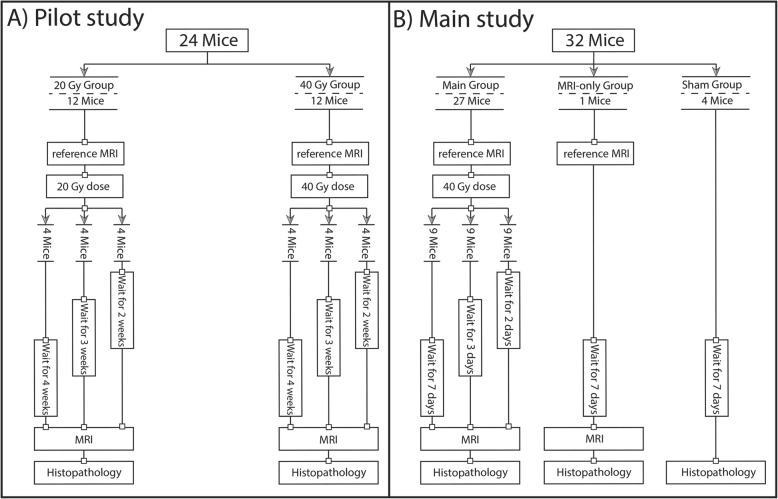
Flow charts of the studies. **a** Pilot study. **b** Main study

The aim of the pilot study was to determine the dose levels and time points, at which histopathology could detect ARIED after a single fraction of radiation dose. We used 20 and 40 Gy dose levels and obtained histopathology at two, three, and 4 weeks after dose delivery, or when mice had to be euthanized for humane endpoints. Five mice were euthanized, four after 8 days (mice 12–15) and one after 24 days (mouse 22) post-irradiation. Twenty-four mice were randomized into the two radiation dose groups (Fig. 1a). There were no control groups.

#### Main study (phase II)

Based on the pilot study outcome, we used a single fraction dose level of 40 Gy in the main study and applied earlier time points: two, three, and 7 days post-irradiation (based on humane endpoints and the severity of ARIED). We randomly divided the 32 mice into the irradiated, MRI-only, and sham groups (Fig. 1b) to allow quality assurance of MRI and histopathology.

### MRI image acquisition

We acquired T2-weighted scans in the axial and sagittal directions on a Biospec 7 T MRI scanner (Bruker Biospin, Ettlingen, Germany) using a 23.0 mm head coil. In the pilot study, a localizer image was performed followed by a T2-weighted rapid acquisition with refocused echo (RARE) in both the axial and sagittal orientations (TR/TE = 2000/39 ms, number of averages = 6, echo train length = 8, matrix size = 192 × 192, voxel size = 0.10 × 0.10 × 0.50 mm^3^). In the main study, the RARE sequences were performed in an axial orientation (TR/TE = 2000/39 ms, with number of averages = 3, echo train length = 8, matrix size = 250 × 200, and voxel size = 0.08 × 0.08 × 1.00 mm^3^) and in a sagittal orientation (TR/TE = 2000/39 ms, with number of averages = 4, echo train length = 8, matrix size = 180 × 250, and voxel size = 0.10 × 0.10 × 0.70 mm^3^. For MR scanning, mice were again anesthetized and placed in the MR scanner. After a localizer image, a T2-weighted scan was performed in both the axial and sagittal orientations. We then euthanized each mouse according to the time points of each group.

### Cone-beam CT imaging and dose delivery

**Fig. 2:**
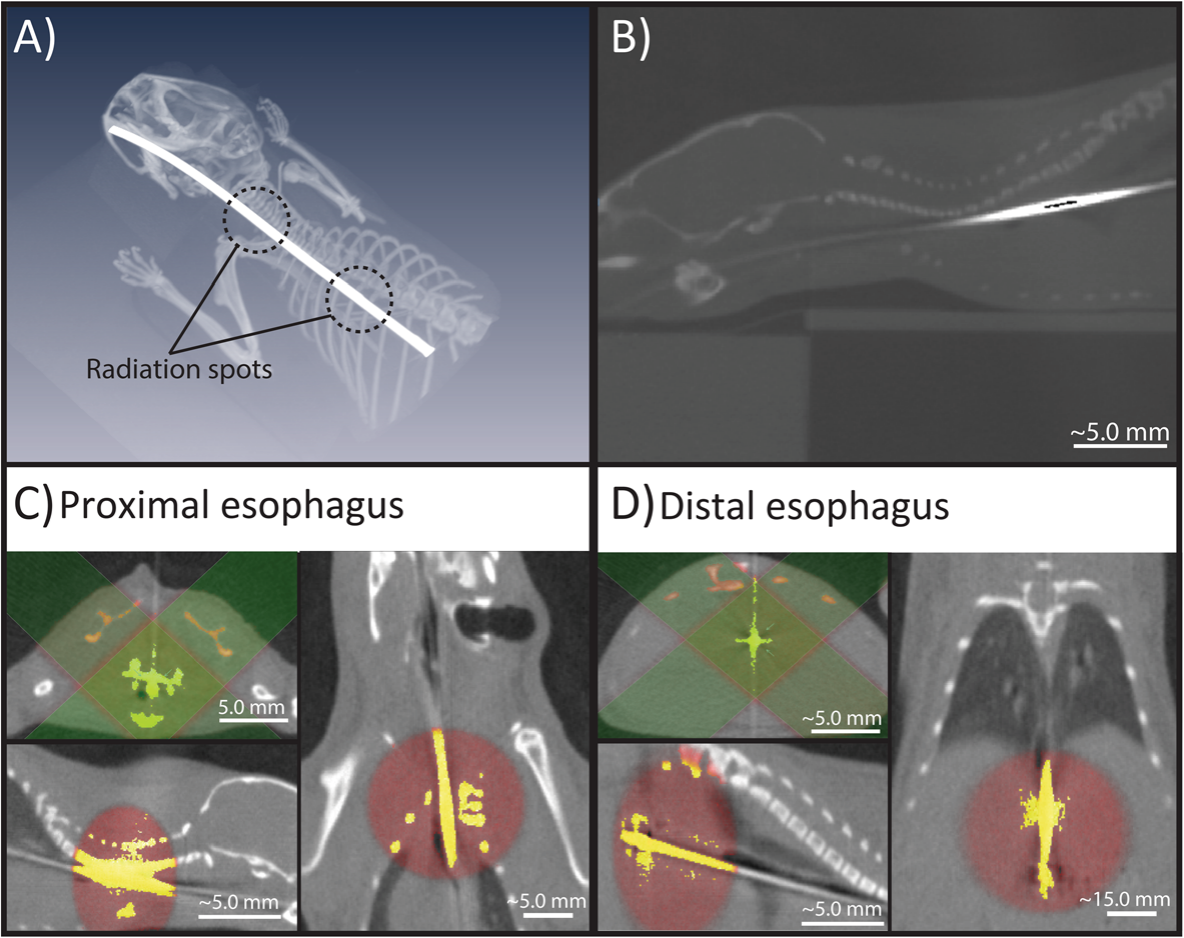
**a** 3D reconstruction of the cone-beam CT illustrating the dose planning and the esophagus (an OCT probe with CT contrast was inserted into the esophagus to differentiate the organ from surrounding tissue). **b** This sagittal cone-beam CT image shows the esophagus with the inserted OCT probe that was filled with CT contrast. **c** and **d** CT images show the radiation fields on the sagittal, coronal and axial planes. The radiation dose was calculated using Monte Carlo simulation

An image-guided small animal irradiation device (X-RAD 225Cx, Precision X-ray Inc., North Branford, CT, USA) was used for cone-beam CT imaging and irradiation. Prior to imaging, we inserted a C7 Dragonfly™ OCT imaging catheter (St. Jude Medical, St. Paul, Minnesota, USA) into mice esophagi to differentiate the esophagus from surrounding tissue in cone-beam CT images for precise dose planning. The probe had an outer diameter of 0.9 mm [15]. To improve its visibility, we filled the catheter with ionic CT contrast (Telebrix Gastro, Guerbert, France), diluted 1:3 in water (Fig. 2) [15]. Mice underwent a scout scan followed by a cone-beam CT to define the irradiation target region and for dose planning [15]. Cone-beam CT imaging was performed with a 2.0 mm Al filter using the following scanning protocol: 40 kV, 0.5 mA, mid gain, 5.0 fps. Irradiation was performed with a 0.3 mm Cu filter with 225 KVp, 13.0 mA, delivering 10 Gy in 255 s. The first irradiation target region was centered on the esophagus around the sixth vertebra of the spinal cord for proximal irradiation. We then delivered a single fraction dose on a 10.0 mm spot centered on the target location using two circular beams. Two lateral beams were used in the pilot study, while in the main study, two orthogonal beams were used (Fig. 2). This change was made to improve consistency with an earlier study [15].

We repeated the same procedure for the distal esophagus by positioning the irradiation spot 10.0 mm further down the esophagus. After irradiation, we took the mice out of the irradiator, retracted the probe, and placed them on a heating pad for recovery.

### Histopathological preparation and analysis

The esophageal specimens were dissected together with a portion of the stomach in order to localize the proximal and distal esophagus. We fixated the esophageal specimens in EAF (ethanol/acetic acid/formaldehyde/saline at 40:5:10:45 v/v), horizontally embedded in paraffin. Following the standard procedure, sagittal sections (at 2.0 μm) of paraffin blocks were stained with hematoxylin and eosin (H&E).

We used a Zeiss Axioskop2 Plus microscope (Carl Zeiss Microscopy, Oberkochen, Germany) and a Zeiss AxioCam HRc digital camera for histopathological analysis and digital microscopic images respectively. Microscopic images were processed using the AxioVision 4 software (Carl Zeiss Vision, Munich, Germany), and organ damage was assessed qualitatively by a pathologist [15].

### Data analysis

**Fig. 3:**
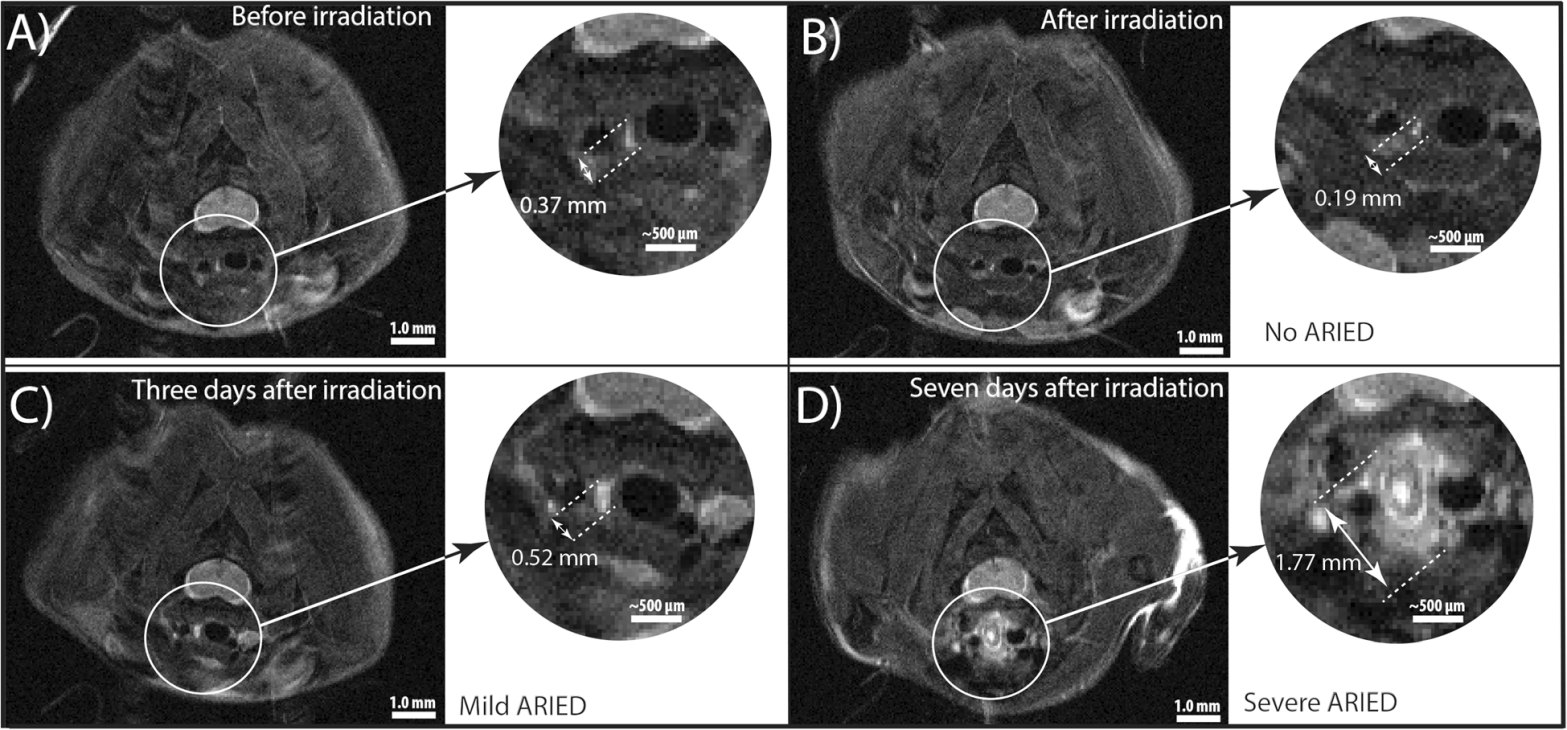
Width measurements of the esophagus and surrounding hyper-intense regions on axial MRI. **a** and **b** are from mouse 8, **c** and **d** are mice 11 and 19 respectively

**Fig. 4:**
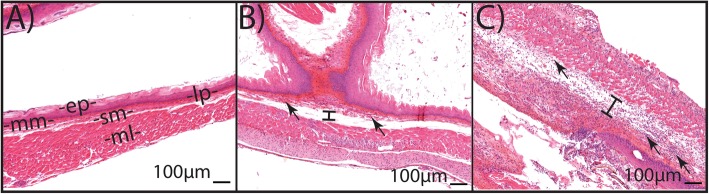
Histopathological illustrations of the esophageal wall layering structure of **a** healthy portion of the esophagus from mouse 3 (Table 1), **b** mild ARIED including mild edema and mild inflammatory infiltration in mouse 10, and **c** severe ARIED with necrosis and severe inflammatory infiltration through whole layers of the wall in mouse 18. Arrows indicate some inflammatory infiltration areas, and edema is shown with the line

For MRI analysis, ARIED was assessed qualitatively by an MRI expert who had no knowledge of the histopathology results. Each image was scored based on the presence of an increased signal intensity and the width of hyperintense area as: no ARIED (no increase in signal intensity or width), mild ARIED (notable increase in signal intensity and/or width), and severe ARIED (large increase in signal intensity and width of hyperintense area around the esophagus). We measured this width from an axial slice in the lower portion of the neck around the sixth vertebra using the ImageJ software (National Institutes of Health), see Fig. 3. We performed width measurements on all mice that underwent MRI before and after irradiation and compared our results with the qualitative findings from histopathology. For statistical analysis, we used repeated measurements of analysis of variance (ANOVA) to evaluate our findings. A *p*-value of < 0.05 is considered as significant. For histopathology analysis, we scored the ARIED based on the spread of the damage, where cases with submucosal ARIED were scored as severe, and others as mild (Fig. 4). ARIED was scored severe in case the histopathology report indicates severe damage in either the proximal or the distal esophagus or both.

## Results

### Pilot study (phase I)

We summarized our MRI and histopathology analyses in Additional file 1: Table S1. Our histopathology results showed limited ARIED in mice that underwent 20 Gy dose delivery (mild ARIED in 18% of all mice). In contrast, 92% of mice that underwent 40 Gy irradiation revealed ARIED in histopathology, of which 64% showed mild damage and 36% severe damage in either proximal or distal portions of the esophagus. Some of the mice reached their humane endpoint before the scheduled time point in the 40 Gy group, and therefore MRI scanning, tissue resection, and histopathology were performed before the scheduled time points. Five mice lost more than 15% of their body weight, of which four were scanned and euthanized after 8 days (mice 12–15) and one after 24 days (mouse 22). These mice were included in the analysis. We excluded one mouse from analysis due to visible necrosis/autolysis in histopathology showing that the mouse died after recovery and prior to the euthanization and resection. The overall sensitivity and specificity of MRI to detect mild or severe ARIED compared to histopathology in the proximal esophagus were 89 and 71% respectively.

Our results showed that a single fraction irradiation of 20 Gy on a 10.0 mm spot was insufficient to induce substantial ARIED in the esophagus of mice up to 2–4 weeks post-irradiation. We therefore did not include the 20 Gy dose group in the main study. Our MRI and histopathology results for the group of 40 Gy dose yielded that severe ARIED occurred in the mice that were euthanized after reaching the humane endpoints at 8 days post-irradiation. Therefore, we moved all our scheduled time points to be in the first week after dose delivery for the main study. Excluding mouse 22 (due to missing data for the distal portion of the esophagus), histopathological analysis after dose delivery of 40 Gy reported ARIED in the proximal portion of the esophagus in seven out of 11 mice (64%) and in distal portion in nine out of 11 mice (82%) (Additional file 1: Table S1).

### Main study (phase II)

#### Qualitative analysis

**Fig. 5:**
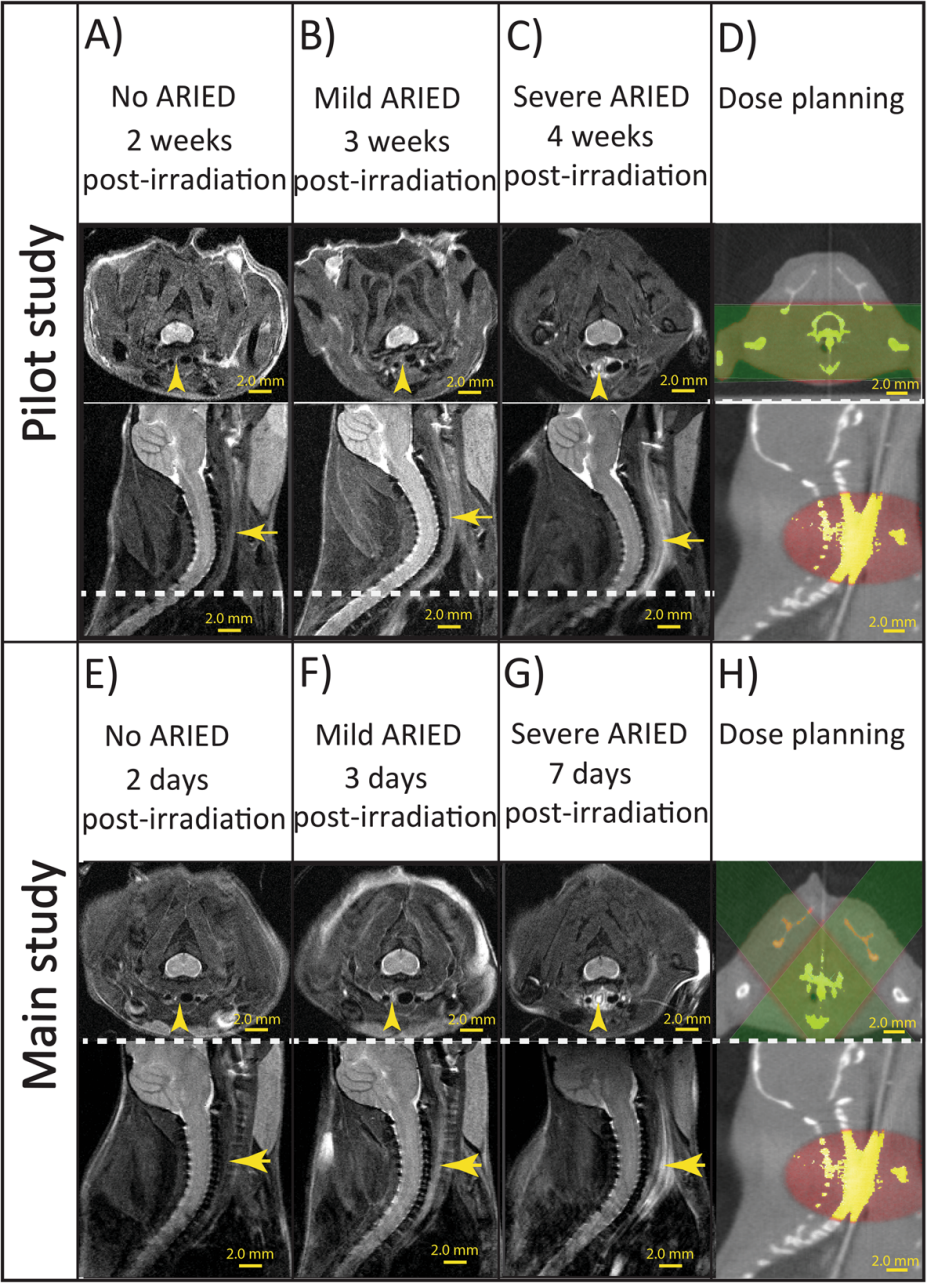
The figure shows MRI images in the axial and sagittal planes of mice with a **a** healthy esophagus, **b** mild ARIED, and **c** severe ARIED at 2, 3, and 4 weeks post irradiation in the pilot study and a **e** healthy esophagus, **f** mild ARIED, and **g** severe ARIED at 2, 3, and 7 days post irradiation in the main study. The cone-beam CT illustrates the dose planning in the axial and sagittal planes in the **d** pilot and **h** main study

Three mice were dropped from the experiment due to incorrect radiation and technical issues with the irradiator. Figure 5 illustrates that we were able to induce ARIED, best visualized in the axial view. Figure 5c and g indicate that because of the large irradiation field of 10.0 mm, neighboring tissues also received a high dose. However, most damage occurred close to the esophagus. The histopathology results are listed in Additional file 1: Table S2. No ARIEDs were reported for the MRI-only and the sham groups in histopathology and MRI analysis. Histopathology found no ARIED, mild ARIED, and severe ARIED in 8, 58, and 33% of all mice respectively. Our MRI analysis of the proximal esophagus showed ARIED in 63% of the irradiated mice (Fig. 5).

Two days post-irradiation only one out of nine mice showed mild ARIED on histopathology and MRI. Three days post-irradiation, mild ARIED in the proximal esophagus was reported in six out of seven mice through histopathological analysis and three out of seven mice with MRI. Histopathological and MRI analysis both indicated mild ARIED in two out of seven mice, from which mice 14 and 15 had mild ARIED on both histopathology and MRI. Seven days post-irradiation, both histopathological and MRI analysis detected severe ARIED for all mice, except in the one case where histopathology data was available. The overall sensitivity and specificity of MRI to detect ARIED compared to histopathology in the main group from 23 mice were 56 and 43% respectively — histopathological results for the proximal portion of the esophagus in mice 6 and 22 were not available (Table 1). However, the in our study MRI detected 100% of severe ARIED. Excluding mice nine and 22, histopathological analysis of mice underwent dose delivery reported damages in the proximal and distal portions of the esophagus in 16 out of 22 mice (73%) and 18 out of 22 mice (82%), respectively (Additional file 1: Table S2).

**Table 1:** Overview of the width of the esophageal lumen (in case of no ARIED) and hyperintense radiation-induced damage regions in MRI images. The numbers represent the largest width of the radiation-induced signal enhancement. Moreover, the table summarized the MRI and histopathology analysis of the proximal esophagus graded in no ARIED, mild ARIED, and severe ARIED. “—” indicates no ARIED and “NA” indicates not available

Width of enhacement around the esophagus (mm)						
Mouse	Group	MRI		Absolute difference (before and post-irradiation)	MRI	Histopathology
		Before Irradiation	Post-irradiation		Proximal	Proximal
1	2 days post-irradiation	0.19	0.05	0.14	–	Mild
2	2 days post-irradiation	0.20	0.15	0.05	–	–
3	2 days post-irradiation	0.17	0.15	0.02	–	Mild
4	2 days post-irradiation	0.14	0.15	0.01	–	Mild
5	2 days post-irradiation	0.23	0.19	0.04	–	Mild
6	2 days post-irradiation	0.47	0.78	0.31	Mild	NA
7	2 days post-irradiation	0.40	0.85	0.45	–	Mild
8	2 days post-irradiation	0.37	0.19	0.18	–	–
9	2 days post-irradiation	0.15	0.25	0.10	–	Mild
10	3 days post-irradiation	0.17	0.15	0.02	–	Mild
11	3 days post-irradiation	0.28	0.52	0.24	Mild	–
12	3 days post-irradiation	0.19	0.52	0.33	Mild	–
13	3 days post-irradiation	0.21	0.42	0.21	Mild	–
14	3 days post-irradiation	0.08	0.80	0.72	Mild	Mild
15	3 days post-irradiation	0.24	1.05	0.81	Mild	Mild
16	3 days post-irradiation	0.15	0.47	0.32	Mild	–
17	7 days post-irradiation	0.11	1.19	1.08	severe	severe
18	7 days post-irradiation	0.19	2.26	2.07	severe	severe
19	7 days post-irradiation	0.45	1.77	1.32	severe	severe
20	7 days post-irradiation	0.36	1.96	1.60	severe	severe
21	7 days post-irradiation	0.39	2.44	2.05	severe	severe
22	7 days post-irradiation	0.34	1.68	1.34	severe	NA
23	7 days post-irradiation	0.40	1.95	1.55	severe	severe
24	7 days post-irradiation	0.18	1.88	1.70	severe	severe
25	MRI-only	0.25	0.15	0.10	–	–

#### Quantitative analysis

Figure 3 shows MR scans of mice before irradiation, as well as 2, 3, and 7 days after irradiation. The MRI analysis was solely based on the proximal portion of the esophagus as the distal portion of the esophagus was not well visible on MRI due to respiration artifacts.

Width measurements of the enhancement are summarized in Table 1. The average healthy esophageal width after irradiation measured on MRI in seven cases based on histopathology scoring results was 0.20 ± 0.10 mm (range: 0.05–0.33 mm, median 0.21 mm).

Similarly, the average width of enhancement around the esophagus for mice with mild ARIED (from nine measurements) and severe ARIED (from seven measurements) were 0.26 ± 0.32 mm (range: 0.01–0.81 mm, median 0.10 mm) and 1.62 ± 0.36 mm (range: 1.08–2.07 mm, median 1.60 mm), respectively. The average enhancement width of the severe radiation-induced damage was 1.36 mm wider than that of the mild damage and 1.41 mm higher than of the esophagus without damage.

**Fig. 6:**
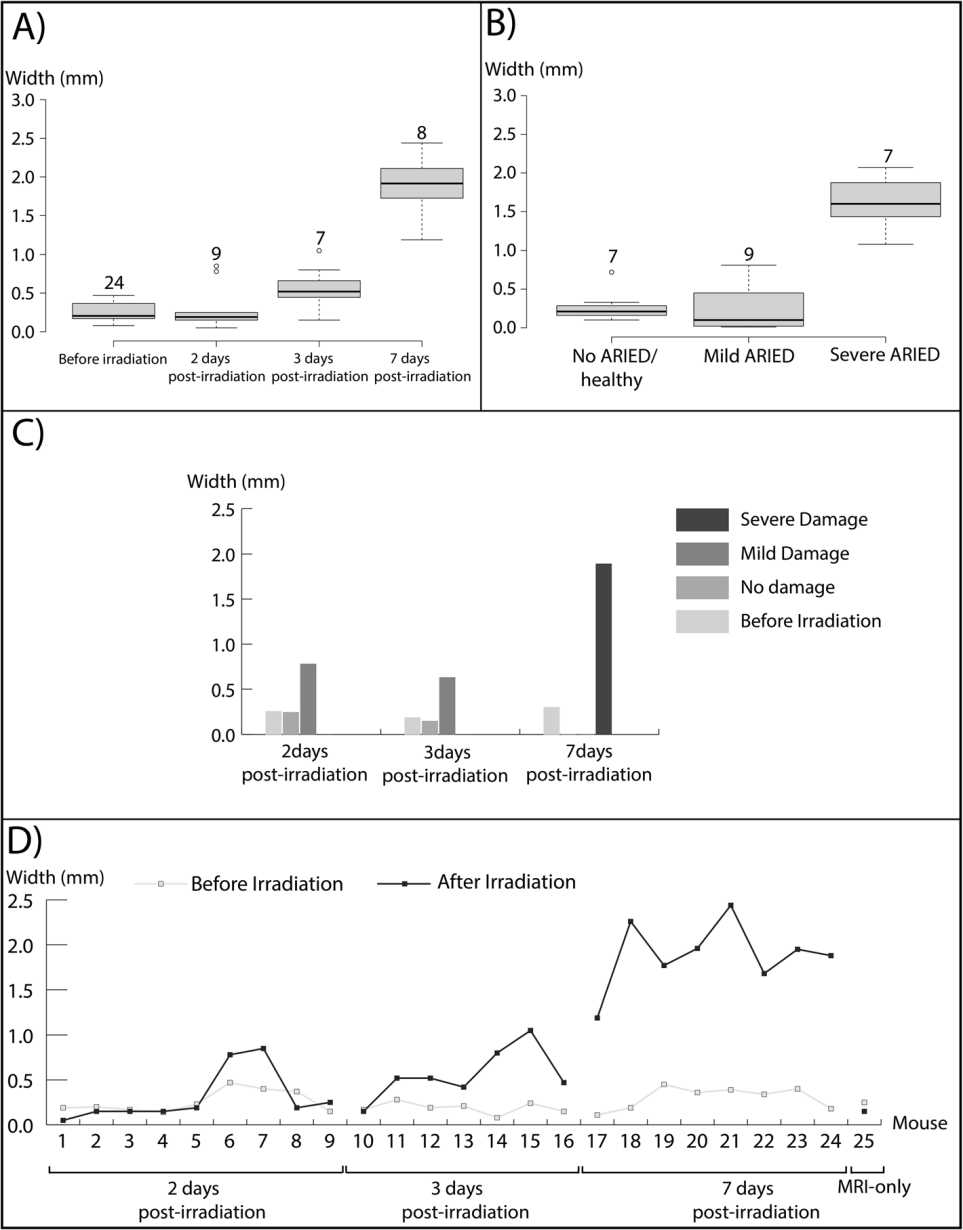
The width of the enhancement region around the esophagus was measured in MRI images for each mouse. Boxes indicate interquartile ranges of the width for **a** various time points after irradiation and **b** no ARIED, mild, and severe ARIED based on histopathological analysis. Horizontal lines show the median values, the circles represent outliners, and the error bars show the range. Plots are also shown for **c** the mean width before and after irradiation as a function of time and damage ranges based on MRI analysis and **d** the width before and after irradiation for all mice

**Fig. 7:**
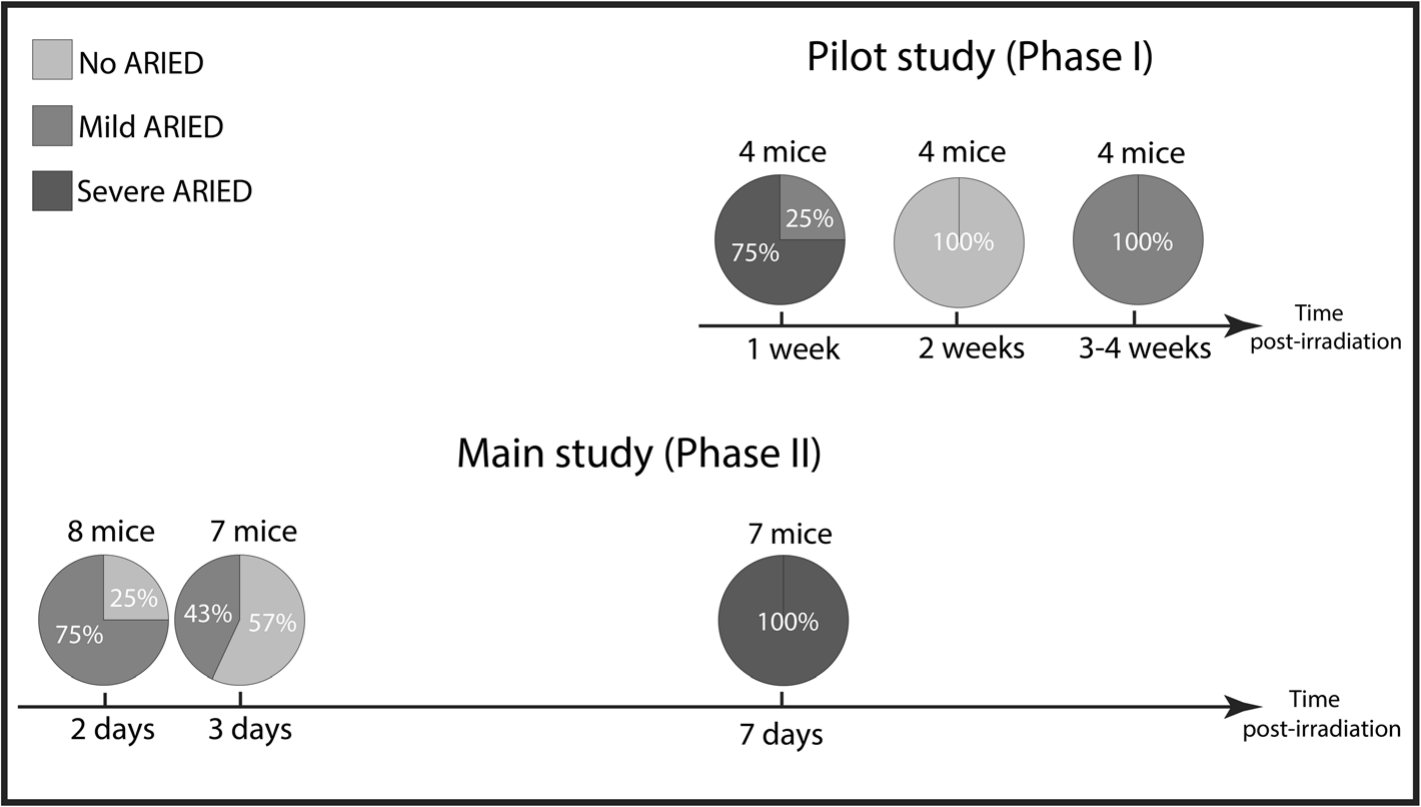
The chart illustrates the occurrence rate of no ARIED, mild ARIED, and severe ARIED as a function of time. The data is based on the histopathological results of the pilot and the main studies for mice receiving 40 Gy dose

While the width difference before and after irradiation was significant for mice with severe ARIED (*p* < 0.00001), it was not significant for mild and no ARIED cases. (*p* = 0.71). Figure 6a and b represent the enhancement width for different time points and the absolute difference of the enhancement width between before and after irradiation for no ARIED/healthy, mild ARIED, and severe ARIED based on histopathological analysis. The average width for different time points and damage ranges — based on MRI findings — is shown in Fig. 6c. The enhancement width of all mice before and after irradiation is shown in Fig. 6d. Figure 7 combines the severity of the ARIED over time from the histopathological analysis of the proximal portion of the esophagus from the pilot and the main studies. Based on our histopathological analysis in the main study (Fig. 6c), the severity of ARIED increases as a function of time until 7 days post-irradiation, which is also confirmed in the histopathological analysis (Fig. 6a). However, if we consider the histopathological analysis of the pilot study for proximal portion for the esophagus (Fig. 7), the ARIED symptoms started to diminish after 1 week and increased again 3 and 4 weeks post-irradiation.

## Discussion

In this study, we investigated the feasibility of MRI to detect and monitor ARIED in the proximal portion of the esophagus in mice that underwent esophageal irradiation. MRI could score ARIED as a change in signal intensity and the width of the hyperintense area around the esophagus, potentially including some acute radiation-induced damage to surrounding tissues. In a pilot study, we showed that a single fraction dose of 20 Gy was insufficient to induce severe ARIED in the mouse esophagus, while a single 40 Gy dose showed considerable ARIED. The pilot study showed that the severity of the ARIED was at a maximum 1 week after irradiation based on weekly measurements. Furthermore, the main study showed that most ARIED occurred within 1 week post-irradiation — between 2 to 7 days timepoints — with mild ARIED occurring from 2 days post-irradiation.

Various clinical studies have investigated the use of MRI to detect and monitor radiation–induced damage to the liver [23–28], myocardium [29, 30], bone marrow [31, 32], the internal architecture of the parotid gland [33–35] and the brain [36–39]. Of course, these studies relate to late responding tissue, and are unlikely comparable to our results for the esophagus. Preclinical studies exist that reported on the use of OCT to detect ARIED in mice [15]. Moreover, other studies used MRI to detect and monitor radiation-induced degenerative changes in articular cartilage and bone in rats [40], progression of radiation-induced necrosis and dose escalation limitations in mouse and rat brains respectively [41, 42], radiation-induced damage in mouse brains [43–46] in xenografts in mice [47, 48], and radiation-induced lung damage in rabbits [49]. However, no studies have reported use of MRI to detect ARIED.

We found limited ARIED after a 20 Gy single fraction dose in the pilot study; which is similar to previous studies on mice and rats in the esophagus and cervical spinal cords respectively [15, 50]. However, our preclinical results contradict clinical experience since the single fraction tolerance of the human esophagus is reported to be 27 Gy based on an α/β ratio at 3 Gy^− 1^ [51]. The discrepancy could potentially be because of differences in the radiobiological sensitivity between humans and mice. Even though the esophageal wall layering is similar between humans and mice, little is known regarding their relative radiobiological sensitivity. For 40 Gy irradiation, the results showed a gradual increase in ARIED from mild to severe by day seven and then a gradual decrease towards mild at 4 weeks. Therefore, MRI and histopathological analysis was most consistent in ARIED identification 1 week post-irradiation, in which most severe ARIED occurred. Based on our analysis in the main study (Fig. 6c), the severity of ARIED increases as a function of time until 7 days post-irradiation, which is also confirmed in the histopathological analysis (Fig. 6a).

The overall sensitivity and specificity of the pilot study were considerably higher than of the main study, which is likely due to substantially longer selected time points of the pilot study. When excluding the observations on day two, the sensitivity of MRI in the main study increased to 90%. Moreover, we also had 20 Gy dose level in the pilot study that induced limited ARIED resulting in more true-negative cases. In general, our main study was designed to induce substantial ARIED. Our results showed that while T2-weighted MRI can visualize severe ARIED, it has a low sensitivity to detect mild ARIED.

In the pilot and the main studies, we gave the same dose to similar locations in the proximal and distal esophagus. However, histopathological analysis yielded slightly more damages in the distal portion of the tissue. Overall, the proximal and distal portions of the esophagus showed damages in 70 and 82% of all mice that underwent 40 Gy dose delivery. This can be due to gastroesophageal refluxes [52] which may sensitize the distal esophagus. Further study is required to show whether indeed the distal portion of the esophagus is more sensitive to irradiation.

The measurements of the esophageal width before and after irradiation showed slight differences for the mice with no ARIED. This could be due to measurement errors but also because the shape of the esophageal lumen is variable over time. We used two orthogonal beams in the main study to resemble the setup of our feasibility study [15]. However, we do not expect much difference in induced damage between lateral and orthogonal beam setups for esophageal damage since the resulting high dose region is similar.

The study design was aimed to find the dose level at which a single dose delivery induces substantial ARIED in the mouse esophagus and to investigate whether MRI can visualize/detect the damage at different time points. The results are limited by necessity because it was unfeasible to combine different time points and dose levels.

A main limitation in detecting ARIED with MRI is the inability to visualize distal portion of the esophagus due to respiration artifacts. With MRI we were only able to visualize the ARIED in the proximal portion of the esophagus, but in humans, we may be able to visualize the distal portion as well. Figure 5 illustrates that ARIED was detected in the same location on the MRI and with a consistent width as the radiation spot diameter of 10.0 mm. Hence, our protocol was appropriate for studying ARIED in mice. We expect that our results can be translated to humans facilitating enhanced detection and monitoring of ARIED, increasing knowledge of ARIED and thereby potentially allowing better healthy tissue sparing while maintaining tumor control. It is crucial to accurately diagnose and monitor ARIED during RT. Our results can be translated to humans, however, clinical studies are required. The advent of the MR guided radiotherapy will greatly facilitate such studies and our work gives an indication of the kind of changes to expect in the esophagus. Furthermore, this study facilitated the comparison between MRI and OCT imaging modalities to detect ARIED [17], affecting future clinical research.

## Conclusion

We investigated whether MRI could detect/monitor ARIED in the mouse esophagus. We delivered dose levels of either 20 or 40 Gy in two 10.0 mm spots. Our qualitative and quantitative results showed the potential of MRI to detect ARIED as a change in signal intensity and the width of hyperintense area around the esophagus. Histopathological analysis of the resected esophagus showed similar results to those from MRI. T2-weighted MRI could visualize severe ARIED, but the sensitivity was low for mild ARIED.

## Supplementary information

**Additional file 1: Table S1.** Histopathology and MRI results showing ARIED in mice for the pilot study where “—” indicates no ARIED. **Table S2.** Histopathology and MRI results showing ARIED in mice for the main study where “—” indicates no ARIED.

## Data Availability

All data generated or analyzed during this study are included in this published article.

## Abbreviations

ANOVA: Analysis of variance
ARIED: Acute radiation-induced esophageal damage
H&E: Hematoxylin and eosin
ICV: Individually ventilated cages
IGRT: Advanced image-guided radiation therapy
MRI: Magnetic resonance imaging
OCT: Optical coherence tomography
PET: Positron emission tomography
RARE: Rapid acquisition with refocused echo
RT: Radiation therapy
WLE: White light endoscopy
