# Supplementary material for: Evaluation of acute esophageal radiation-induced damage using magnetic resonance imaging: a feasibility study in mice
Source: Radiat Oncol. 2019 Oct 30;14:188. doi: 10.1186/s13014-019-1396-8 (PMC6822441; doi:10.1186/s13014-019-1396-8)
Supplement: Supplementary file 1 — Additional file 1: Table S1. Histopathology and MRI results showing ARIED in mice for the pilot study where “—” indicates no ARIED. Table S2. Histopathology and MRI results showing ARIED in mice for the main study where “—” indicates no ARIED. [file 13014_2019_1396_MOESM1_ESM.docx]

**Additional file 1**

**Table S1.** Histopathology and MRI results showing ARIED in mice for the pilot study where “—” indicates no ARIED.

| **Group** | **Termination time** | **Mouse** | **MRI** | **Histopathology** | |
| --- | --- | --- | --- | --- | --- |
|  |  |  | **Proximal esophagus** | **Proximal esophagus** | **Distal esophagus** |
| **20 Gy group** | **After 2 weeks** | **1** | — | — | — |
|  |  | **2** | — | — | Mild inflammatory infiltrations in the lamina propria of the mucosa.   \|  \| \| --- \| |
|  |  | **3** | — | — | — |
|  | **After 3 weeks** | **4** | — | — | — |
|  |  | **5** | — | — | — |
|  |  | **6** | — | — | — |
|  |  | **7** | — | — | — |
|  | **After 4 weeks** | **8** | — | Some edematous changes in the submucosa. | — |
|  |  | **9** | — | — | — |
|  |  | **10** | — | — | — |
|  |  | **11** | — | — | — |
| **40 Gy group** | **After 1 week** | **12** | Severe ARIED | Local severe lesions of necrosis of the mucosa with sharp demarcations, accompanied by inflammatory infiltrations through whole layers of the wall, edema in the submucosa, and dilation of the esophagus.   \|  \| \| --- \| | Local similar lesions as the ones described in the proximal part of this esophagus. Severe inflammatory and degenerative lesions are also seen in the gastric mucosa.   \|  \| \| --- \| |
|  |  | **13** | Severe ARIED | Local severe lesions of necrosis of the mucosa with sharp demarcations in the middle* part of the esophagus, accompanied by inflammatory infiltrations through whole layers of the wall, and edema in the submucosa.   \|  \| \| --- \| | Local severe lesions of necrosis of the mucosa with sharp demarcations in the middle* part of the esophagus, accompanied by inflammatory infiltrations through whole layers of the wall, and edema in the submucosa.   \|  \| \| --- \| |
|  |  | **14** | Severe ARIED | Local severe lesions of necrosis of the mucosa with sharp demarcations, accompanied by inflammatory infiltrations and edema in the mucosa and submucosa.   \|  \| \| --- \| | Mild inflammatory infiltrations in the submucosa.   \|  \| \| --- \| |
|  |  | **15** | Mild to severe ARIED | Local inflammatory infiltrations in the lamina propria of the mucosa and submucosa. | Local severe lesions of necrosis of the mucosa with sharp demarcations, accompanied by inflammatory infiltrations through whole layers of the wall, and edema in the submucosa. |
|  | **After 2 weeks** | **16** | Mild ARIED | — | Mild inflammatory infiltrations in the lamina propria of the mucosa.   \|  \| \| --- \| |
|  |  | **17** | Mild ARIED | — | — |
|  |  | **18** | Mild ARIED | — | Local inflammatory infiltration through mucosa to serosa of whole layers of the wall.   \|  \| \| --- \| |
|  |  | **19** | Mild to severe ARIED | — | Mild inflammatory infiltrations in the lamina propria of the mucosa.   \|  \| \| --- \| |
|  | **After 3 weeks** | **20** | Mild ARIED | Focal mild inflammatory infiltrations in the submucosa of the middle* part of the esophagus. | Focal mild inflammatory infiltrations in the submucosa of the middle* part of the esophagus. |
|  |  | **21** | Mild ARIED | Local mild edematous changes in the submucosa. | Not Available |
|  | **After 24 days** | **22** | Mild ARIED | Local edematous changes in the submucosa. | — |
|  | **After 4 weeks** | **23** | Mild ARIED | Mild edematous changes in the submucosa. | Mild edematous changes in the submucosa. |

^* We considered proximal and distal ARIED for such cases due to difficulties to identify the exact location of the damaged regions in histopathology specimens.^

**Table S2.** Histopathology and MRI results showing ARIED in mice for the main study where “—” indicates no ARIED.

| **Group** | **Termination time** | **Mouse** | **MRI** | **Histopathology** | |
| --- | --- | --- | --- | --- | --- |
|  |  |  | **Proximal esophagus** | **Proximal esophagus** | **Distal esophagus** |
| **40 Gy group** | **After 2 days** | **1** | — | At the very beginning of the esophagus (at the level of larynx), focal degenerative lesions of the muscularis externa. | Local degenerative lesions of the muscularis externa, accompanied by inflammatory infiltrations with extension to the submucosa and to the lamina propria of the mucosa. ­­­­ |
|  |  | **2** | — | — | Local necrotic lesions of the muscularis externa, accompanied by inflammatory infiltrations in submucosa and adventitia. |
|  |  | **3** | — | Local degenerative/necrotic lesions of the muscularis externa at the middle* part of the esophagus, accompanied by edema and mild inflammatory infiltrations in subcutis and adventitia. | |
|  |  | **4** | — | Small and mild degenerative lesions of muscularis externa. | — |
|  |  | **5** | — | Local necrotic lesions of the muscularis externa, accompanied by inflammatory infiltrations with extension to the submucosa. | Local necrotic lesions of the muscularis externa, accompanied by inflammatory infiltrations with extension to the submucosa. |
|  |  | **6** | — | Local lesions of necrosis of the muscularis externa with inflammatory infiltrations.   \|  \| \| --- \| | Local lesions of necrosis of the muscularis externa with mild inflammatory infiltrations.   \|  \| \| --- \| |
|  |  | **7** | — | — | — |
|  |  | **8** | — | Small degenerative lesions of the muscularis externa.   \|  \| \| --- \| | Focal lesions of necrosis of the muscularis externa with mild inflammatory infiltrations in the submucosa and adventitia.   \|  \| \| --- \| |
|  |  | **9** | Mild ARIED | Not available | Local degenerative lesions of the muscularis externa. |
|  | **After 3 days** | **10** | — | Small and mild degenerative lesions in the muscularis externa. | Local degenerative lesions of the muscularis externa, accompanied by mild inflammatory infiltrations. |
|  |  | **11** | Mild ARIED | — | Focal degenerative lesions of the muscularis externa. |
|  |  | **12** | Mild ARIED | — | — |
|  |  | **13** | Mild ARIED | — | Local lesions of inflammatory infiltrations in the submucosa, accompanied by dilation of the blood vessels. |
|  |  | **14** | Mild ARIED | Mild edematous changes with mild inflammatory infiltrations in the submucosa.   \|  \| \| --- \| | - Local degenerative lesions of the muscularis externa accompanied by inflammatory infiltration with extension to submucosa.  - Local inflammatory infiltrations in the adventitia with dilation of blood vessels.   \|  \| \| --- \| |
|  |  | **15** | Mild ARIED | Multifocal lesions of necrosis of the muscularis externa accompanied by inflammatory infiltration with extension to submucosa.   \|  \| \| --- \| | Rather large lesions of necrosis of the muscularis externa accompanied by inflammatory infiltration with extension to submucosa as well as adventitia.   \|  \| \| --- \| |
|  |  | **16** | Mild ARIED | — | Small degenerative lesions of the muscularis externa.   \|  \| \| --- \| |
|  | **After 7 days** | **17** | Severe ARIED | Severe necrotic changes of the mucosa at the middle* and distal parts of the esophagus, accompanied by severe edematous changes and inflammatory infiltrations in the submucosa with extensions to muscularis externa and adventitia. | Severe necrotic changes of the mucosa at the middle* and distal parts of the esophagus, accompanied by severe edematous changes and inflammatory infiltrations in the submucosa with extensions to muscularis externa and adventitia. |
|  |  | **18** | Severe ARIED | **­­**Severe necrotic changes of the mucosa, accompanied by edema and inflammatory infiltrations in the submucosa with extensions to muscularis externa and adventitia. | **­­**Severe necrotic changes of the mucosa, accompanied by edema and inflammatory infiltrations in the submucosa with extensions to muscularis externa and adventitia. |
|  |  | **19** | Severe ARIED | Severe necrotic changes of the mucosa, accompanied by edema and inflammatory infiltrations in the submucosa with extensions to muscularis externa and adventitia. | Severe necrotic changes of the mucosa, accompanied by edema and inflammatory infiltrations in the submucosa with extensions to muscularis externa and adventitia. |
|  |  | **20** | Severe ARIED | Severe necrotic changes of the mucosa, accompanied by edema and inflammatory infiltrations in the submucosa with extensions to muscularis externa and adventitia. | Severe necrotic changes of the mucosa, accompanied by edema and inflammatory infiltrations in the submucosa with extensions to muscularis externa and adventitia. |
|  |  | **21** | Severe ARIED | Severe necrotic changes of the mucosa, accompanied by edema and inflammatory infiltrations in the submucosa with extensions to muscularis externa and adventitia. | Severe necrotic changes of the mucosa, accompanied by edema and inflammatory infiltrations in the submucosa with extensions to muscularis externa and adventitia. |
|  |  | **22** | Severe ARIED | Not available | Severe necrotic changes of the mucosa, accompanied by edema and inflammatory infiltrations in the submucosa with extensions to muscularis externa and adventitia. |
|  |  | **23** | Severe ARIED | Severe necrotic changes of the mucosa, accompanied by edema and inflammatory infiltrations in the submucosa with extensions to muscularis externa and adventitia. | — |
|  |  | **24** | Severe ARIED | Severe necrotic changes of the mucosa, accompanied by edema and inflammatory infiltrations in the submucosa with extensions to muscularis externa and adventitia. | Severe necrotic changes of the mucosa, accompanied by edema and inflammatory infiltrations in the submucosa with extensions to muscularis externa and adventitia. |
| **MRI-only** |  | **25** | — | — | — |
| **Sham group** |  | **26** | Not available | — | — |
|  |  | **27** | Not available | — | — |
|  |  | **28** | Not available | — | — |
|  |  | **29** | Not available | — | — |

^* We considered proximal and distal ARIED for such cases due to difficulties to identify the exact location of the damaged regions in histopathology specimens.^
